# Vitamin D as a modulator of pain and inflammation in postmenopausal females with burning mouth syndrome

**DOI:** 10.22514/jofph.2025.008

**Published:** 2025-03-12

**Authors:** Jeong-Hyun Kang

**Affiliations:** ^1^Samsung Advanced Institute for Health Sciences & Technology, Sungkyunkwan University, 06355 Seoul, Republic of Korea; ^2^Clinic of Oral Medicine and Orofacial Pain, Institute of Oral Health Science, Ajou University School of Medicine, 16499 Suwon, Republic of Korea; ^3^Department of Dental Education, Yonsei university, College of Dentistry, 03722 Seoul, Republic of Korea

**Keywords:** Vitamin D, Inflammation, Burning mouth syndrome, Pain, Oral health-related quality of life

## Abstract

**Background**: Vitamin D has roles in neurological, hormonal and immunological processes, affecting various pain disorders and related comorbidities. The aim of this study was to investigate relationship between vitamin D levels and clinical features in postmenopausal females with burning mouth syndrome (BMS). **Methods**: This retrospective, cross-sectional study reviewed clinical and laboratory data from 144 postmenopausal females with BMS. Laboratory tests measured 25-(OH) hydroxyvitamin D, hematic components and inflammatory markers. Participants were categorized by serum levels of 25-(OH) hydroxyvitamin D, as deficient (<20 ng/mL), inadequate (20–30 ng/mL), and adequate (>30 ng/mL). Pain intensity and oral health-related quality of life were assessed using visual analog scale (VAS), McGuill Pain Questionnaire (MPQ) and Oral Health Impact Profile-49 (OHIP-49). **Results**: Pain intensity and oral health-related quality of life were associated with serum vitamin D levels. Hemoglobin, folic acid and high-sensitivity C-reactive protein (hs-CRP) concentrations varied among groups. Serum 25-(OH) hydroxyvitamin D levels showed negative correlation with VAS, MPQ sensory, MPQ affective, MPQ evaluative and OHIP-49 scores, indicating lower pain intensity and suffering with higher vitamin D levels. Additionally, iron levels were negatively related to VAS score, while folic acid levels were negatively associated with OHIP-49 score. Serum 25-(OH) hydroxyvitamin D levels were negatively correlated with hs-CRP levels. **Conclusions**: These findings suggest significant interactions between 25-(OH) hydroxyvitamin D levels and pain intensity and suffering and oral health-related quality of life, indicating its therapeutic potential for postmenopausal BMS patients.

## 1. Introduction

Burning mouth syndrome (BMS) is defined as a chronic pain condition featured by persistent pain in the oral mucosa, manifesting as burning or dysaesthetic sensations without visible oral lesions or other objective signs [1]. This condition generally persists for over 2 hours daily and lasts for more than 3 months [1]. BMS primarily affects peri- or postmenopausal females, and its causes are diverse. Potential contributing factors include local nerve trauma, parafunctional habits, hyposalivation, hormonal changes caused by menopause, and psychological factors such as anxiety and depression [2, 3, 4].

Recent studies have emphasized the significance of both central and peripheral neuropathic etiology in BMS development [5]. Evidences from quantitative sensory tests, blink tests, tongue biopsies, functional brain magnetic resonance imaging, positron emission tomography, and genetic analysis support the peripheral and central neuropathic etiological background of BMS [5]. Repetitive local irritations to the oral mucosa, oral parafunctional habits, hyposalivation and alterations in salivary composition can lead to peripheral neuropathic changes in the oral mucosa [6, 7, 8]. In addition, signs of decreased central nervous system inhibition, such as impaired brainstem reflex habituation [9], and reduced striatal dopamine function [10, 11] have been observed in patients with BMS, implying the involvement of central neuropathic mechanisms. Consequently, various pathophysiological mechanisms or therapeutic approaches targeting both central and peripheral neuropathic modulation and neuroinflammation have been proposed for BMS management [2, 12, 13]. Therefore, understanding these complex pathophysiological factors is crucial when seeking effective therapeutic targets for BMS.

Approximately 50% of Korean females aged 50–69 were found to have vitamin D deficiency or inadequate intake [14]. Vitamin D is widely recognized for its role in calcium homeostasis and bone metabolism. However, it also influences anatomical, neurological, hormonal and immunological processes, affecting various pain disorders and related comorbidities [15, 16]. Vitamin D can inhibit neuroinflammation by reducing proinflammatory cytokines and increasing anti-inflammatory cytokines [17, 18]. Furthermore, recent studies have suggested a link between low vitamin D levels and the incidence of chronic neuropathic pain conditions, such as painful diabetic peripheral neuropathy and postherpetic neuralgia [19, 20]. In addition, previous studies demonstrated the therapeutic effects of vitamin D_3_ on cold allodynia and heat hyperalgesia in rat models of neuropathic pain [21], and improvements in functional recovery and myelination after vitamin D_3_ administration [22]. Given that neuroinflammation and central or peripheral neuropathic pain mechanisms are well-known components of BMS pathophysiology [5, 23], the anti-inflammatory properties and therapeutic effects of vitamin D on neuropathic pain disorders could potentially alleviate BMS symptoms.

Increased prevalence of vitamin D deficiency was reported in patients with BMS [24]. Despite the neuropathic nature of BMS, sparse studies have specifically examined the relationships between pain intensity and suffering in BMS and vitamin D levels. Hence, the aim of the present study was to investigate the relationship between vitamin D levels and clinical features, including pain intensity and suffering in postmenopausal females with BMS.

## 2. Materials and methods

### 2.1 Participants

This cross-sectional, retrospective study reviewed clinical and laboratory data from 144 postmenopausal females with BMS, who visited a tertiary hospital in South Korea between March 2018 to February 2024. All patients reported experiencing subjective burning sensations or dysesthesia in the oral cavity, without objective clinical signs, for more than 2 hours daily and lasting over 3 months [1]. Each patient underwent laboratory tests and completed a comprehensive interview and self-administered questionnaires from previous study [25] to evaluate pain characteristics. BMS is a multifactorial condition influenced by various etiological factors, including hormonal imbalance, psychological factors, local nerve trauma [2, 3, 4]. Previous studies have highlighted that hormonal imbalance or fluctuations in peri-menopausal females are among the primary contributors to BMS [25]. To minimize these influences, ensure sample homogeneity, and account for the predominance of BMS in menopausal and post-menopausal females, only female postmenopausal BMS patients who were more than 2 years post-menopause were included in this study [26]. Patients interview and clinical evaluations, including oral examination and psychological tests were conducted during initial visits by a single orofacial pain specialist (JHK). Laboratory tests and salivary flow rate measurements were performed during follow-up visits, after 8 hours fasting.

Participants were excluded if they were taking cytotoxic drugs or immunosuppressants, had a history of chemotherapy, had moderate to severe, life threatening anemia (hemoglobin levels less than 10 g/dL) [27], uncontrolled diabetes, thyroid disorder, autoimmune diseases such as systemic lupus erythematosus, dermatomyositis, Sjogren’s syndrome, fibromyalgia, or rheumatoid arthritis, or other types of orofacial neuropathic pain disorders including trigeminal neuralgia, a history of beam radiation and/or radioisotope treatment in the head and neck region, or were unable to communicate effectively. Initially, 269 postmenopausal females with BMS were included. After excluding 5 patients with a history of malignancy, 2 with rheumatoid arthritis, 3 with moderate anemia, 2 with a history of trigeminal neuralgia, and 113 participants, who did not report burning sensation a final total of 144 BMS patients remained in the study (Fig. 1).

**Fig. 1.** Flow of study participant selection.

Participants were categorized into three groups based on their serum 25-(OH) hydroxyvitamin D levels, as deficient (<20 ng/mL), inadequate (20–30 ng/mL), and adequate (>30 ng/mL). This classification aligns with clinical guidelines for assessing vitamin D status [28, 29].

The study protocol was approved by the Institutional Review Board of the Ajou University Hospital (AJIRB-DB-2024-082) in accordance with the Declaration of Helsinki. Informed consent was waived by Institutional Review Board due to retrospective study design.

### 2.2 Clinical and psychological evaluation and outcome variables

The clinical evaluation procedures for the participants included oral examinations, salivary flow rate measurements, and a simplified psychological evaluation using the Symptom Checklist-90-Revised (SCL-90-R) to determine levels of depression and anxiety [30]. Patients interview, clinical evaluations, including oral examination and psychological tests were conducted during initial visits, while salivary flow rate measurements were performed during follow-up visits. The salivary flow rate assessment involved collecting unstimulated whole saliva (UWS) by having participants drool into a tube for 10 minutes. Stimulated whole saliva (SWS) was collected during mechanical stimulation by chewing 1 g of gum base. The saliva collected in the first 2 minutes was discarded and that collected in the subsequent 5 minutes was collected for analysis. The salivary flow rate was expressed in mL/min. UWS and SWS measurements were taken after participants refrained from eating, drinking or toothbrushing at least 1 hour.

The primary outcome was pain intensity, assessed by a visual analog scale (VAS) ranging from 0 to 10, with 10 indicating the worst imaginable pain. Secondary outcomes included the McGuill Pain Questionnaire (MPQ) and the Oral Health Impact Profile-49 (OHIP-49). The MPQ evaluates sensory, affective, evaluative and miscellaneous aspects of pain using a Likert scale, comprising 78 words categorized into three major classes, including sensory, affective and evaluative dimensions, which are further into 20 subclasses [31]. The sensory dimension measures the sensory qualities of the pain experience, while the affective dimension describes emotional qualities such as tension, fear and autonomic properties associated with the pain experience. Additionally, the evaluative dimensions reflect the subjective overall intensity of the total pain experience. Meanwhile, the OHIP-49 assesses the impact of oral health on quality of life through 49 items across seven domains [32]. Functional limitation assesses difficulties with speaking and pronunciation. Physical pain measures the level of pain or discomfort in the mouth or facial areas. Psychological discomfort reflects concerns or self-consciousness about oral health. Physical disability addresses challenges with eating certain foods due to oral health issues. Psychological disability captures feeling of sadness or dissatisfaction related to oral health. Social disability measures social avoidance or embarrassment, and handicap reflects a reduction in life satisfaction or enjoyment. Each item is rated on a 5-Likert scale from 0 to 4, with higher scores indicating a more significant negative impact on oral health-related quality of life.

### 2.3 Biochemical assessment and determination of serum vitamin D levels

Venous blood samples were collected from all participants for comprehensive analysis and differential diagnosis. Laboratory tests for differential diagnosis were conducted during follow-up visits after a minimum of 8 hours of fasting at follow-up visit. Previous report suggest that several hematic factors and micronutrients may play roles in the occurrence of secondary BMS [33]. The tests included complete blood counts with leukocyte differential and hematinic-related components such as iron, ferritin, vitamin B_12_, total iron-binding capacity (TIBC), unsaturated iron-binding capacity (UIBC), and folic acid. Folate, vitamin B_12_ are indicator for erythropoiesis and DNA synthesis, while TIBC and UIBC reflects the iron-binding capacity. Ferritin serves as an indicator of iron storage. In addition, levels of micronutrients, including magnesium, zinc, copper and manganese were measured. Serum inflammatory markers, including high-sensitivity C-reactive protein (hs-CRP) and erythrocyte sedimentation rate (ESR), were also determined to rule out inflammatory diseases.

### 2.4 Statistical analysis

Sample size calculations were based on a two-sided type I error rate of 5% and a power of 80%. The proportion between vitamin D deficiency, inadequacy, and adequacy were estimated from previous report, which indicated that among 50–59-year-old Korean females, approximately, 55% had vitamin D deficiency, 35% had inadequacy, and 10% had adequacy [14]. Power analysis determined that a total sample size of 144 participants, comprising 34 with vitamin D deficiency, 42 with vitamin D inadequacy, and 68 with adequate vitamin D levels would provide a statistical power of 93.4% at a 0.05 significance level with an effect size of 0.4 for one-way analysis of variance (ANOVA). The effect size was calculated by dividing the differences in group means by the standard deviation of the pooled data from the VAS.

The normality of data was assessed using a combination of Shapiro-Wilk normality test and normal Quantile-Quantile plot (Q-Q plot), which supported the use of parametric statistical methods. Fig. 2 presents the histogram and Q-Q plot illustrating the serum vitamin D data distribution. To compare differences in demographic features, symptom duration, clinical feature distribution and levels of serum markers among the groups, one-way ANOVA and chi-square tests were applied for continuous and categorical variables, respectively. Pearson’s correlation analysis was conducted to reveal the associations between variables.

**Fig. 2.** Normal distribution of the data. (A) Histogram of serum vitamin D level distribution. (B) Q-Q plot.

To identify the interdependent influence of vitamin D on BMS symptoms, each variable, significantly associated with serum 25-(OH) hydroxyvitamin D levels in the univariate comparison, along with basic demographic factors, was incorporated into the multivariate linear regression as confounders. The main outcome variables were included pain characteristics and features, as well as the level of oral health-related quality of life. These were assessed using VAS, MPQ sensory, MPQ affective, MPQ evaluative, MPQ miscellaneous scores and the OHIP-49 score, respectively. The primary independent variable was serum level of 25-(OH) hydroxyvitamin D. Model 1 was age-adjusted, whereas Model 2 was adjusted for age, symptom duration, UWS and SCL-90-R scores for depression and anxiety. The multivariate Model 3 was further adjusted for VAS and OHIP-49 scores for when McGuill scores were the outcome variable, McGuill scores and OHIP-49 scores when VAS was the outcome variable, and VAS and McGuill scores when OHIP-49 was the outcome variable. All tests were two-sided, and *p*-values less than 0.05 were considered statistically significant.

## 3. Results

### 3.1 Demographic and clinical features of the participants

Age (*p* = 0.017), postmenopausal period (*p* = 0.029), pain intensity and suffering levels varied significantly according to serum vitamin D levels. No significant differences were detected in symptom duration, unstimulated whole saliva (UWS), and stimulated whole saliva (SWS) in relation to serum 25-(OH) hydroxyvitamin D level. The distribution of background underlying diseases, including hypertension, diabetes mellitus, dyslipidemia, osteoporosis, cardiovascular diseases, cerebral infarction, liver disease and asthma did not exhibit significant differences among groups. However, variables related to pain intensity, including VAS (*p* = 0.017), MPQ sensory score (*p* = 0.032), MPQ affective score (*p* = 0.002), MPQ evaluative (*p* = 0.030), and those related to oral health-related quality of life, such as the OHIP-49 scores (*p* = 0.026), were significantly different among the groups. The tongue was the most frequent pain site across all three groups, in that approximately 91.0% of the participants reporting it as the main discomfort site, and approximately 29.2% experienced taste change as well as burning symptoms. The distribution of pain sites, characteristics of intraoral discomfort other than burning sensation and psychological factors did not show statistical difference in relation to vitamin D levels, except throbbing sensation. Throbbing sensation was more prevalent in patients with inadequate vitamin D serum levels (*p* = 0.004) (Table 1).

**Table 1.** Clinical characteristics of participants with BMS.

		Vitamin D deficient, (n = 34)	Vitamin D inadequate, (n = 42)	Vitamin D adequate, (n = 68)	*p *value	*Post-hoc*
Age		63.3 ± 11.4	55.8 ± 12.0	58.7 ± 10.8	0.017*	deficiency-inadequate
Postmenopausal period (mon)		18.0 ± 10.1	12.4 ± 9.0	12.7 ± 8.2	0.029*	deficiency-adequate
Underlying diseases (Yes, %)^†^						
	Hypertension	7 (20.6)	9 (21.4)	7 (10.3)	0.212	
	Diabetes mellitus	6 (17.6)	2 (4.8)	6 (8.8)	0.159	
	Dyslipidemia	9 (26.5)	9 (21.4)	11 (16.2)	0.460	
	Osteoporosis	1 (2.9)	2 (4.8)	6 (8.8)	0.458	
	Cardiovascular diseases	1 (2.9)	3 (7.1)	2 (2.9)	0.518	
	Cerebral infarction	0	0	1 (1.5)	0.570	
	Liver diseases	0	1 (2.4)	0	0.294	
	Asthma	0	0	1 (1.5)	0.570	
Symptom duration (mon)		20.5 ± 30.3	19.9 ± 26.8	17.2 ± 24.1	0.796	
Salivary flow rate						
	UWS (mL/min)	0.23 ± 0.17	0.22 ± 0.23	0.22 ± 0.28	0.982	
	SWS (mL/min)	1.22 ± 2.29	0.89 ± 0.46	0.90 ± 0.46	0.419	
Pain intensity						
VAS		7.24 ± 2.00	7.57 ± 1.90	6.49 ± 2.04	0.017*	inadequate-adequate
MPQ						
	Sensory	17.2 ± 5.4	13.5 ± 7.5	11.8 ± 10.5	0.032*	deficiency-adequate
	Affective	5.48 ± 3.75	2.64 ± 2.68	2.92 ± 3.11	0.002*	deficiency-inadequate, inadequate-adequate
	Evaluative	2.96 ± 2.44	1.59 ± 1.22	2.00 ± 1.69	0.030*	deficiency-inadequate
	Miscellaneous	2.38 ± 3.54	3.05 ± 3.11	3.13 ± 3.97	0.703	
OHIP-49		95.7 ± 43.2	86.4 ± 34.9	66.4 ± 47.0	0.026*	deficiency-adequate
Pain site^†^						
	Tongue only	21 (61.8)	25 (59.5)	40 (58.8)	0.314	
	Tongue and others	13 (38.2)	12 (28.6)	20 (29.4)		
	Others only	0	5 (11.9)	8 (11.8)		
Other pain characteristics except burning (Yes, %)^†^						
	Throbbing	4 (11.8)	15 (35.7)	8 (11.8)	0.004*	
	Stinging	5 (14.7)	9 (21.4)	11 (16.2)	0.698	
	Paresthesia	1 (2.9)	1 (2.4)	3 (4.4)	0.837	
	Taste change	11 (32.4)	15 (35.7)	16 (23.5)	0.353	
	Dry mouth	10 (29.4)	10 (23.8)	11 (16.2)	0.282	
Psychological Evaluation (SCL-90-R)						
	Depression	47.0 ± 9.2	48.0 ± 9.0	46.3 ± 9.6	0.667	
	Anxiety	48.7 ± 10.0	48.3 ± 9.7	47.4 ± 9.4	0.667	

UWS, unstimulated whole saliva; SWS, stimulated whole saliva; VAS, visual analog scale; MPQ, McGuill Pain Questionnaire; OHIP-49, Oral Health Impact Profile-49; SCL-90-R, Symptom Checklist-90-Revised. Descriptive values are shown as mean ± SD. SD: standard deviation. ^†^Descriptive values are shown as N (%, percentage by column). Data obtained from one-way ANOVA and Chi-square test with Bonferonni’s post-hoc analysis. *p < 0.05, by one-way ANOVA and Chi-square test.

### 3.2 Laboratory findings of participants

Micronutrients and certain hematic factors, such as iron, UIBC, TIBC, vitamin B_12_ and ferritin did not appear to be influenced by vitamin D levels, however serum levels of folic acid and inflammatory marker hs-CRP seemed to be affected by vitamin D levels. No significant differences were detected in the serum levels of hematic factors and micronutrients, such as iron, UIBC, TIBC, vitamin B_12_, ferritin, magnesium, zinc, copper and manganese. However, serum concentrations of hemoglobin (*p* = 0.047) and folic acid (*p* = 0.009) were significantly different among the groups. Inflammatory markers also showed significant interactions with 25-(OH) hydroxyvitamin D levels, with serum hs-CRP level (*p* = 0.003) showing significant differences among the groups. On the other hand, the other inflammatory marker, ESR did not show significance differences (Table 2).

**Table 2.** Laboratory findings of participants.

	Vitamin D deficient (n = 34)	Vitamin D inadequate (n = 42)	Vitamin D adequate (n = 68)	*p *value	*Post-hoc*
25-(OH) hydroxyvitamin D (ng/mL)	13.8 ± 3.9	25.5 ± 2.6	43.8 ± 18.4	<0.001**	deficiency-inadequate, deficiency-adequate, inadequate-adequate
ESR (mm/h)	10.70 ± 7.80	9.15 ± 6.28	10.10 ± 7.40	0.675	
hs-CRP (mg/dL)	0.22 ± 0.40	0.06 ± 0.12	0.08 ± 0.12	0.003*	deficiency-inadequate, deficiency-adequate
Hemoglobin (g/dL)	12.9 ± 1.9	13.6 ± 1.3	13.4 ± 1.0	0.047*	
Iron (µg/dL)	88.1 ± 38.2	88.0 ± 33.9	95.2 ± 33.5	0.449	
UIBC (µg/dL)	230.2 ± 62.4	236.3 ± 47.5	218.1 ± 56.3	0.227	
TIBC (µg/dL)	319.2 ± 43.9	324.2 ± 45.8	313.7 ± 46.6	0.498	
Folic acid (µg)	10.7 ± 5.1	14.2 ± 6.7	15.0 ± 7.1	0.009*	deficiency-adequate
Vitamin B_12_	1161.3 ± 1413.3	989.1 ± 397.6	1042.4 ± 520.8	0.642	
Ferritin (µg/L)	137.6 ± 107.4	133.5 ± 90.1	103.5 ± 97.7	0.084	
Magnesium (mg/dL)	2.18 ± 0.14	2.24 ± 0.18	2.20 ± 0.17	0.259	
Zinc (mcg/dL)	86.2 ± 13.8	83.7 ± 13.7	82.7 ± 13.3	0.478	
Copper (mcg/dL)	102.7 ± 19.4	101.1 ± 13.7	107.0 ± 17.2	0.189	
Manganese (µg/L)	11.0 ± 3.8	10.1 ± 2.8	11.4 ± 3.4	0.149	

ESR, erythrocyte sedimentation rate; hs-CRP, high-sensitivity C-reactive protein; UIBC, unsaturated iron-binding capacity; TIBC, total iron-binding capacity. Descriptive values are shown as mean ± SD. Data obtained from one-way ANOVA and Chi-square test with Bonferonni’s post-hoc analysis. *p < 0.05, **p < 0.001 by one-way ANOVA test.

### 3.3 Relationships between vitamin D levels, clinical manifestations and hematic features

Serum vitamin D levels appeared to significantly impact pain intensity and oral health-related quality of life and were associated with various hematological factors. Pearson’s correlation analysis demonstrated that serum 25-(OH) hydroxyvitamin D levels exhibited significant interactions with parameters related to pain intensity and oral health-related quality of life, including VAS (*r*^2^ = −0.194, *p* = 0.020), MPQ sensory (*r*^2^ = −0.359, *p * < 0.001), MPQ affective (*r*^2^ = −0.237, *p* = 0.024) and OHIP-49 scores (*r*^2^ = −0.329, *p * < 0.001). Several hematic factors also showed significant relationships with clinical variables, such as the iron level with the VAS score (*r*^2^ = −0.202, *p* = 0.016) and the folic acid concentration with the OHIP-49 score (*r*^2^ = −0.250, *p* = 0.019). The serum 25-(OH) hydroxyvitamin D level was significantly correlated with hs-CRP level (*r*^2^ = −0.195, *p* = 0.019) (Table 3).

**Table 3.** Correlations among serum levels of vitamin D, hemoglobin, iron, folic acid, ESR, hs-CRP and pain intensity and oral health-related quality of life in BMS patients.

	VAS	MPQ (Sensory)	MPQ (Affective)	MPQ (Evaluative)	MPQ (Miscellaneous)	OHIP-49	Hemoglobin	Iron	Folic acid	ESR	hs-CRP
25-(OH) hydroxyvitamin D	−0.194*	−0.359**	−0.237*	−0.227*	0.020	−0.329**	0.097	−0.043	0.100	−0.103	−0.195*
VAS		0.299**	0.090	0.004	0.097	0.292**	0.018	−0.202*	−0.115	0.138	0.137
MPQ (Sensory)			0.607**	0.390**	0.367**	0.426**	0.067	−0.061	−0.081	0.035	0.036
MPQ (Affective)				0.496**	0.434**	0.189	0.007	−0.041	−0.117	0.069	0.140
MPQ (Evaluative)					0.124	0.089	−0.087	0.012	−0.031	−0.178	−0.171
MPQ (Miscellaneous)						0.276*	−0.011	−0.095	−0.104	0.018	−0.051
OHIP-49							0.077	−0.130	−0.250*	−0.022	0.137
Hemoglobin								0.369**	−0.102	−0.143	−0.083
Iron									0.053	−0.097	−0.117
Folic acid										0.143	−0.048
ESR											0.425**

VAS, visual analog scale; MPQ, McGuill Pain Questionnaire; OHIP-49, Oral Health Impact Profile-49; ESR, erythrocyte sedimentation rate; hs-CRP, high-sensitivity C-reactive protein. The bolded numbers indicate values that are statistically significant. *p < 0.05, **p < 0.001 by Pearson’s correlation analysis.

After adjusting for related confounders, serum vitamin D levels continued to exhibit significant influences. In Model 1, after adjusting for age, VAS (*p* = 0.021), MPQ sensory (*p * < 0.001), MPQ affective (*p* = 0.024), MPQ evaluative (*p* = 0.035) and OHIP-49 (*p* = 0.002) scores showed significant relationships with serum 25-(OH) hydroxyvitamin D levels. In Model 2, after adjusting for other confounders such as age, symptom duration, UWS, and psychological factors, including anxiety and depression, 25-(OH) hydroxyvitamin D still exhibited significant associations with the VAS (*p* = 0.007), MPQ sensory (*p* = 0.002) and OHIP-49 (*p* = 0.007) scores. In Model 3, significant relationships remained for VAS (*p* = 0.012) scores with serum 25-(OH) hydroxyvitamin D levels (Table 4).

**Table 4.** Associations between serum levels of 25-(OH) hydroxyvitamin D and pain intensity and oral-health related quality of life in postmenopausal females with BMS.

Outcome variable	Model 1 (Age adjusted)		Model 2		Model 3	
	OR (95% CI)	*p* value	OR (95% CI)	*p* value	OR (95% CI)	*p* value
VAS	−0.022 (−0.040–−0.003)	0.021*	−0.026 (−0.046–−0.007)	0.007*	−0.037 (−0.066–−0.009)	0.012*
MPQ (Sensory)	−0.179 (−0.218–0.084)	<0.001**	−0.165 (−0.269–−0.061)	0.002*	−0.087 (−0.195–0.020)	0.108
MPQ (Affective)	−0.046 (−0.084–0.045)	0.024*	−0.042 (−0.084–0.001)	0.055	−0.013 (−0.008–0.030)	0.246
MPQ (Evaluative)	−0.024 (−0.047–−0.002)	0.035*	−0.022 (−0.047–0.002)	0.069	−0.020 (−0.047–0.006)	0.135
MPQ (Miscellaneous)	0.004 (−0.040–0.048)	0.857	0.007 (−0.040–0.055)	0.766	0.018 (−0.039–0.074)	0.538
OHIP-49	−0.838 (−0.843–0.728)	0.002*	−0.744 (−1.282–−0.205)	0.007*	−0.336 (−0.928–0.256)	0.261

VAS, visual analog scale; MPQ, McGuill Pain Questionnaire; OHIP-49, Oral Health Impact Profile-49; OR, odd ratio; CI, confidential interval. Estimated from multivariate linear regression Model 1 with age to estimate ORs an 95% CIs. The multivariate Model 2 was adjusted for age, symptom duration, UWS, scores for depression and anxiety. The multivariate Model 3 was further adjusted for VAS and OHIP-49 scores for when McGuill scores were the outcome variable, McGuill scores and OHIP-49 scores when VAS was the outcome variable, and VAS and McGuill scores when OHIP-49 was the outcome variable. *p < 0.05, **p < 0.001 by multivariate linear regression.

## 4. Discussion

BMS is a chronic pain disorder of the oral mucosa with no objective clinical signs and has a complex etiology involving both central and peripheral neuropathic mechanisms [1, 2, 4, 5, 6, 7, 8, 9, 10, 11]. Numerous studies have demonstrated the relevance of these mechanisms in BMS development, leading to therapeutic strategies targeting neuropathic modulation [2, 12, 32]. Vitamin D, which reduced neuroinflammation by modulating proinflammatory cytokine levels [17, 18], has been applied to manage various neuropathic pain conditions [19, 20, 21, 22]. Despite the established neuropathic basis of BMS, research which particularly has examined the association between vitamin D levels and pain intensity or suffering in BMS patients are sparse. Therefore, the aim of the present study was to elucidate the link between vitamin D levels and pain intensity or suffering in postmenopausal females suffering from BMS.

The main finding of the present study is the potential role of vitamin D in influencing pain intensity and suffering in postmenopausal females with BMS. Our results indicated that individuals in the vitamin D deficiency and inadequacy groups exhibited higher pain intensity, as indicated by VAS scores, MPQ scores, as well as lower oral health-related quality of life compared to those in the adequate group. Additionally, serum vitamin D levels were significantly negatively corelated with pain intensity and oral health-related quality of life, including VAS, MPQ sensory, affective, evaluative and OHIP-49 scores. The MPQ captures various aspects of pain, with the MPQ sensory and evaluative dimension reflecting sensory characteristics and overall appraisal of the pain, respectively, while MPQ affective dimension capturing the emotional quality of the pain experience. Serum vitamin D levels appeared to influence all of these dimensions. Moreover, the significant relationship between VAS and serum vitamin D levels persisted even after full adjustment in the multivariate linear regression analysis. Therefore, vitamin D may be linked not only with to pain intensity, indicated by VAS and MPQ sensory and evaluative scores, but also with specific sensory and emotional aspects of pain, as reflected by MPQ affective scores in BMS patients.

A previous study has reported that nearly 15% of the patients with BMS showed lower vitamin D_3_ levels [24] and another study found that 74.5% of patients with BMS had hypovitaminosis D, which was significantly related to subjective oral dryness and candidiasis [34]. Additionally, increased levels of ransient receptor potential cation channel subfamily V member 1 (TRPV1) and nerve growth factor-positive nerve fibers have been reported in the tongue tissue of BMS patients compared to healthy controls, suggesting that abnormal TRPV1 expression may be involved in pain modulating mechanisms of BMS [35]. TRPV1 is activated by various inflammatory stimuli and mediators, which can exacerbate pain signaling [36]. Recent studies have proposed that vitamin D can act as a partial agonist of the TRPV1 and its anti-inflammatory properties may impact TRPV1 activities [17, 18, 37]. By acting on TRPV1, vitamin D may help regulate excessive TRPV1 activation and mitigate the inflammatory responses, potentially providing relief from chronic pain conditions such as BMS.

The anti-inflammatory effects of vitamin D are well-documented. Recent studies have shown that vitamin D regulates the functions of monocytes and macrophages by increasing the production of antimicrobial peptides [38, 39]. It promotes anti-inflammatory effects in macrophages by boosting Interleukin-10 (IL-10) production and reducing the production of proinflammatory cytokines such as IL-2, IL-6 and tumor necrosis factor-alpha (TNF-α) [38, 39, 40, 41]. Previous reports have also detected increased levels of inflammatory cytokines in the serum and saliva of patients with BMS [42, 43]. The preceding results demonstrated that patients with BMS and vitamin D deficiency exhibited significantly higher hs-CRP levels than the other groups. These findings suggest that systemic inflammatory reactions may contribute to the occurrence and progression of BMS. Vitamin D has the potential to alleviate the systemic inflammatory process underlying BMS, thereby reducing pain and improving the quality of life of patients with BMS.

Deficiencies in hematic factors such as hemoglobin, iron, vitamin B complex, ferritin and folate are well-known contributors to the development of burning or stinging symptoms in the oral cavity [44, 45, 46]. In this study, serum iron levels showed a significant relationship with pain intensity and folic acid levels demonstrated a significant association with oral health-related quality of life. Iron and hemoglobin deficiencies may cause subtle atrophy of the oral epithelium, resulting in a burning sensation in the tongue and oral discomfort [47]. A previous study proposed the role of vitamin D in anemia [48]. Vitamin D directly suppresses the expression of hepcidin mRNA, an antimicrobial protein, and reduces proinflammatory cytokine production [49]. In addition, evidence shows that vitamin D may protect against anemia by supporting erythropoiesis [50]. Therefore, low serum vitamin D levels may affect erythropoiesis and increase the severity of inflammation, leading to a burning sensation in the tongue or oral mucosa. Even though, patients with uncontrolled severe anemia were excluded from this study, the relationship between hematic factors, vitamin D, and the pathophysiology of BMS still remains a consideration.

The present study investigated the potential role of vitamin D in influencing pain intensity and suffering among postmenopausal females with BMS. However, there are several limitations. Firstly, the cross-sectional study design and absence of a control group inhibited deriving conclusive evidence regarding the pathophysiology and therapeutic effects of vitamin D on BMS. Secondly, even though the majorities of BMS patients were peri- or post-menopausal females, this study focused exclusively on postmenopausal females, limiting the generalizability of the findings to younger females and males. Thirdly, a previous study found that among 50–59-year-old Korean females, approximately 55% had vitamin D deficiency, 35% had inadequacy, and 10% had adequacy [14]. In contrast, in this study, the rates of deficiency, inadequacy, and adequacy were 23.6%, 29.2% and 47.2%, respectively. While the previous study targeted the general population nationwide, this study focused on participants who visited tertiary hospital in a major city. As a result, the sample likely included individuals with relatively higher income and education levels, which could have influenced their medication conditions, including vitamin D levels. Finally, CRP and ESR levels can be influenced not only by systemic diseases such as rheumatoid arthritis, but also by other local factors like periodontitis, sinusitis, upper respiratory infections which can cause low-grade inflammation. However, the assessment of these factors has not been conducted, aside from the evaluation of mucosal lesions. Therefore, to confirm the therapeutic effects of vitamin D on BMS, future prospective randomized controlled trials including both sexes and diverse age ranges are warranted.

## 5. Conclusions

The etiology of BMS is multifactorial, involving a complex interplay of peripheral and central neuropathic mechanisms, inflammatory reactions, and interactions with hematic factors. Vitamin D, with its anti-inflammatory properties, neuroprotective capacities, and role in erythropoiesis, can potentially influence pain mechanisms in patients with BMS. The preceding findings suggest significant interactions between vitamin D levels and pain intensity and suffering, indicating its therapeutic potential for BMS, particularly in postmenopausal females. A comprehensive understanding of the role of vitamin D, the pathways modulating pain and the pathophysiological background of BMS is essential for appropriate diagnosis and management of BMS.
